# Aortic valve repair for isolated right coronary leaflet prolapse

**DOI:** 10.1016/j.xjtc.2022.02.031

**Published:** 2022-03-03

**Authors:** Arnar Geirsson, Clarence H. Owen, Robert S. Binford, Rochus K. Voeller, Christopher R. Burke, Jeffrey D. McNeil, Lawrence M. Wei, Vinay Badhwar, J. Scott Rankin

**Affiliations:** a Department of Cardiac Surgery, Yale University School of Medicine, New Haven, Conn; b Cone Health, Greensboro, NC; c Overlake Hospital Medical Center, Belleview, Wash; d Department of Cardiothoracic Surgery, University of Minnesota, Minneapolis, Minn; e Department of Cardiac Surgery, University of Washington, Seattle, Wash; f Cardiovascular and Thoracic Surgeons of Austin, Austin, Tex; g Department of Cardiovascular and Thoracic Surgery, West Virginia University, Morgantown, WVa

**Keywords:** aortic valve insufficiency, aortic valve repair, aortic ring annuloplasty, AI, aortic insufficiency, AVr, aortic valve repair, RCP, right coronary leaflet prolapse

## Abstract

**Objectives:**

Isolated right coronary leaflet prolapse is a common cause of nonaneurysmal aortic insufficiency, but can rarely occur in patients with proximal aortic aneurysms. Standardized techniques for routine autologous repair of this disorder are presented.

**Methods:**

Most aortic valve leaflet prolapse is isolated to the right coronary leaflet, with hypertension and annular dilatation being contributory. Echocardiographically, a posteriorly eccentric aortic insufficiency jet together with “fracture” of the right leaflet tip are diagnostic. Primary repair includes internal geometric ring annuloplasty to downsize and reshape the annulus, together with central plication of the prolapsing leaflet. Thickened, scarred, or retracted noduli are released using an ultrasonic aspirator. The goal is to achieve equivalent coaptation heights of ≥8 mm for all 3 leaflets.

**Results:**

Three videos of 6 cases are provided to illustrate these techniques. In the first, 3 patients are shown with classic isolated right leaflet prolapse. In the second and third videos, alternative pathologies are presented for contrast. Applying the reconstructive approaches of geometric ring annuloplasty, leaflet plication, and ultrasonic nodular release, excellent early and late repair outcomes are obtainable in most patients.

**Conclusions:**

The combination of aortic ring annuloplasty, central leaflet plication, and ultrasonic nodular release allows routine and standardized repair of right coronary leaflet prolapse, either isolated or concomitant with aneurysm surgery.

**Figure:**
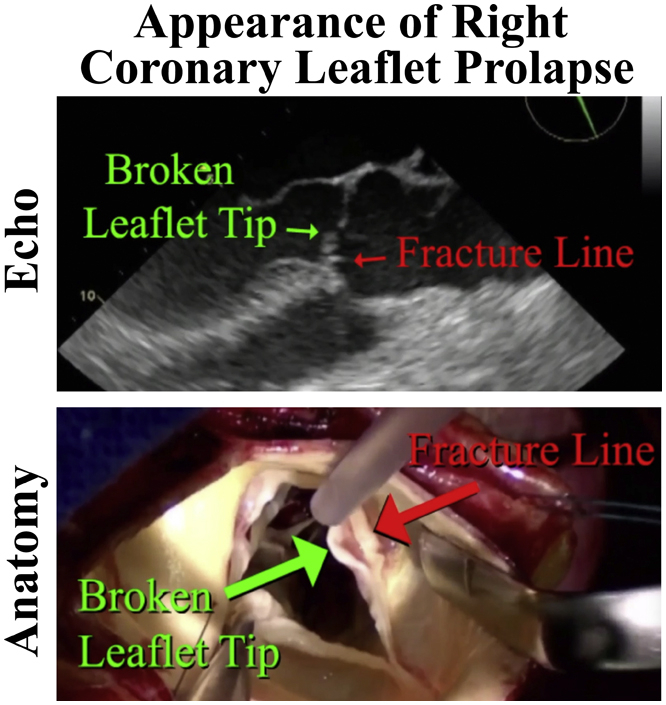
Clinical characteristics of isolated right coronary leaflet prolapse.

**Central Message**

Echocardiographic and anatomic illustrations of isolated right coronary leaflet prolapse demonstrate a “fracture line” in the body of the leaflet and a “broken” leaflet tip, prolapsing into the ventricle.

**Perspective**

Right coronary leaflet prolapse is a common cause of aortic insufficiency. Usually the defect is isolated but rarely can accompany proximal aortic aneurysms. The annular dilatation and leaflet prolapse are readily repaired with a combination of geometric ring annuloplasty, central leaflet plication, and nodular release when needed.

Leaflet prolapse of the aortic valve without the presence of an aneurysm or bicuspid defect is relatively common, and right coronary leaflet prolapse (RCP) is a frequently encountered cause of aortic insufficiency (AI).^1^ In RCP, hypertension and annular dilatation are initiating factors that cause the leaflets to lose their central support of coaptation. Because the aortic valve annulus is elliptical,^2^ and the right coronary leaflet attaches to the flat side of the ellipse, the right coronary leaflet has a larger radius of curvature (Figure 1). Hypothetically, pressure from the aorta on the larger curvature of the uncoapted right leaflet produces higher stress and predisposes the leaflet tip to “fracture” and prolapse. The AI jet is characteristically posterior and eccentric onto the undersurface of anterior mitral leaflet. The pathology is consistent and distinct, and thus, might be described with new terminology, such as the leaflet tip being “broken” with an associated transverse “fracture line” (Figure 2). This report illustrates aortic valve repair (AVr) for isolated RCP in 3 typical patients, and for comparison, other variations of aortic leaflet prolapse and nodular retraction are presented as a contrast.

**Figure 1:**
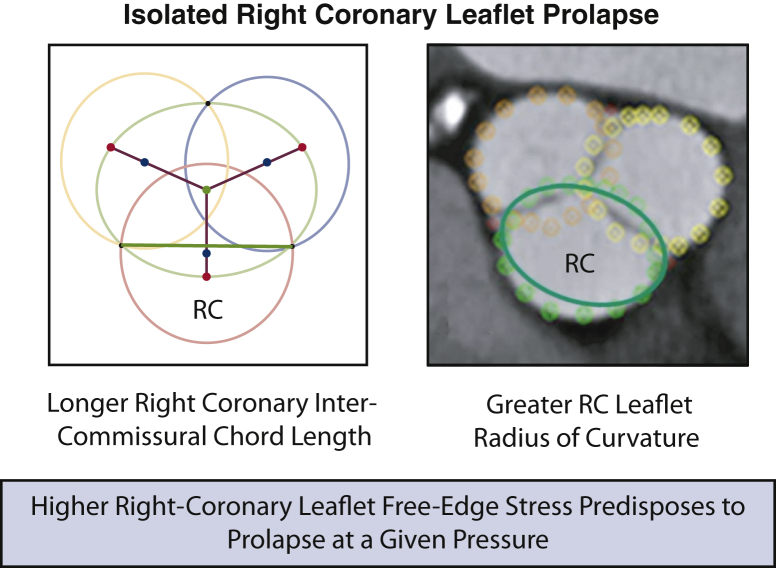
Because of the elliptical nature of the aortic valve annulus,^2^ the right coronary leaflet (*RC*) has a greater radius of curvature, higher leaflet stress, and hypothetically, a predisposition to “fracture.”

**Figure 2:**
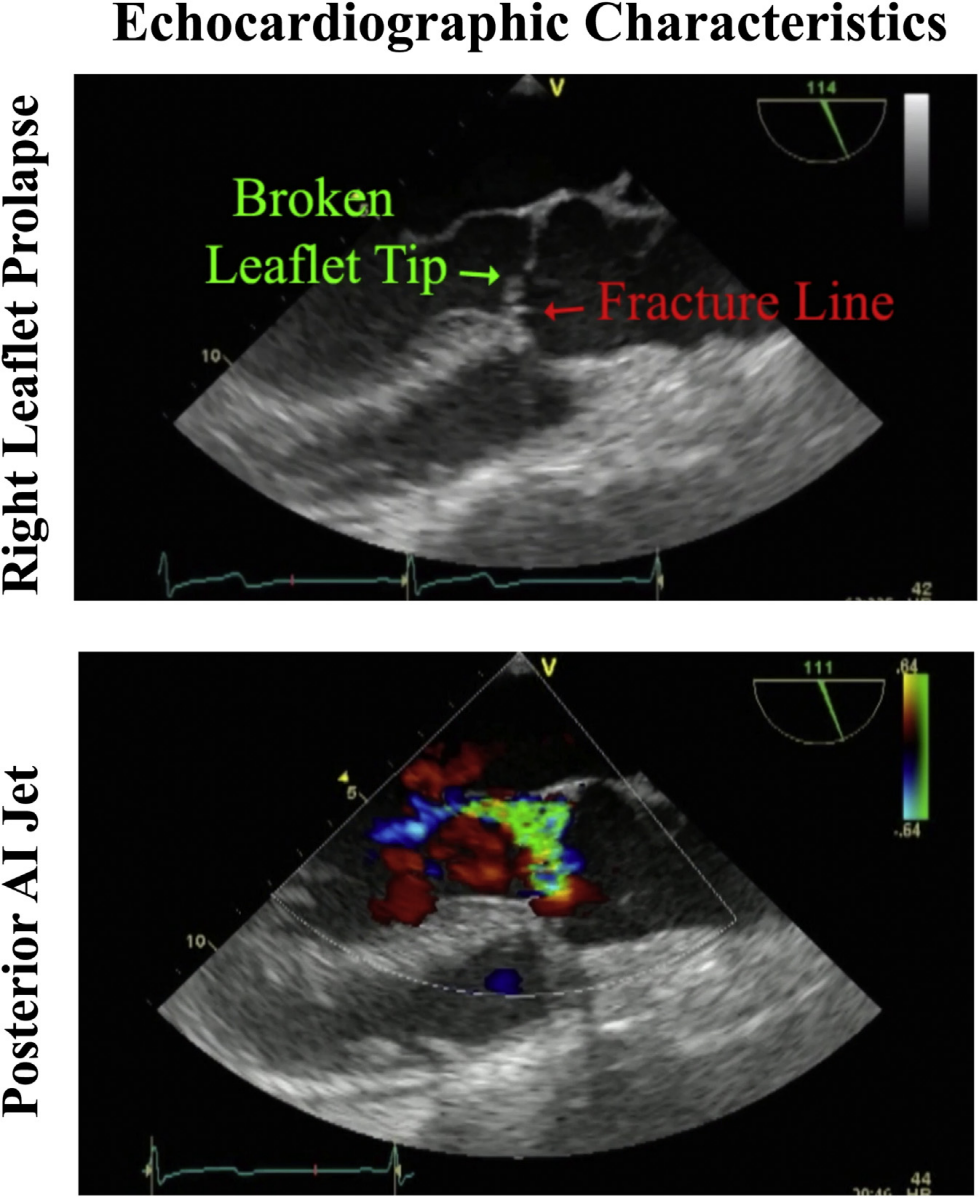
Typical echocardiogram of a patient with isolated right coronary leaflet prolapse showing a “broken” leaflet tip, a leaflet “fracture line,” and a posterior aortic insufficiency (*AI*) jet.

## Methods

The institutional review board approved this study and publication of these data; protocol number 2005016064; May 29, 2020 to May 28, 2025. Patient written consent for publication of the study data was waived by the institutional review board for retrospective analysis of deidentified clinical information.

### Clinical Description

The proposed “syndrome” of RCP is frequently encountered in clinical practice and comprises two-thirds of nonaneurysmal leaflet prolapse cases.^1^ Clinical features include: (1) late middle-aged men with trileaflet valves and a history of hypertension, (2) no significant aortic aneurysm, (3) aortic annular dilatation in the range of 27 to 29 mm, (4) a very eccentric posterior AI jet, and (5) right coronary leaflet pathology consisting of a transverse “fracture line” with a “broken” tip prolapsing into the ventricle (Figure 2). Techniques for AVr in this disorder have been detailed elsewhere,^3^ and consist of geometric ring annuloplasty sized as (leaflet free-edge length)/(1.5) to downsize and reshape the annulus. The right leaflet prolapse is corrected with central plication to raise the right cusp coaptation height to the same level as the left and noncoronary leaflets. Possibly because of the chronic AI, scarring and retraction of the Noduli Arantii are common, requiring ultrasonic nodular release. This clinical and anatomic syndrome is highly specific, and because extensive leaflet calcification and/or retraction are rare, almost all RCP valves can be repaired—although a modest learning curve exists for leaflet plication. In Video 1, 3 typical cases of isolated RCP are presented to illustrate the consistent pathologic features and repair techniques for this clinical syndrome.

### Case Video Summaries

#### Video 1

**Figure 3:**
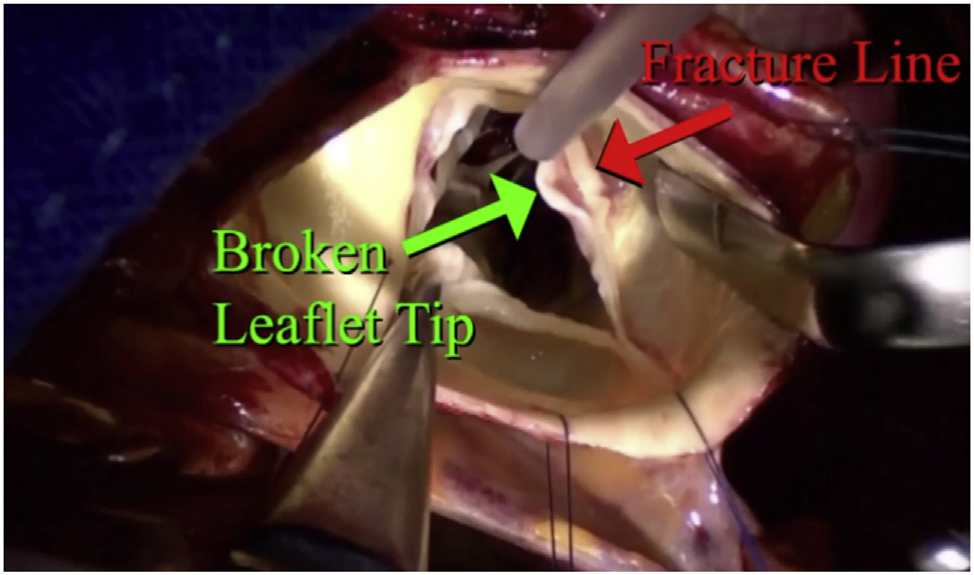
Typical appearance of isolated right coronary leaflet prolapse with a “broken” leaflet tip and “fracture line.”

The first patient was a 68-year-old man with heart failure, severe AI, an aortic sinus diameter of 46 mm, and a 27-mm aortic valve annulus on echocardiography. On echocardiography, the RCP and posterior AI jet were evident (Figure 2). A circular aortotomy was performed 1 cm above the commissural tops (Video 1). A “broken”, prolapsing right coronary leaflet tip was present, with a transverse “fracture line” (Figure 3). Hegar annular diameter was 28 mm, and using ball sizers, the leaflets sized to 21 and 23 mm, using the formula: required ring diameter = (leaflet free-edge length)/(1.5).^3^ A trileaflet aortic annuloplasty ring was selected (HAART 300; BioStable Science and Engineering), and because of the need for leaflet plication, the ring was downsized to 21 mm. The ring posts were sutured to the 3 subcommissural triangles using Cabrol-like horizontal mattress sutures (Video 1). Two ring-looping sutures were placed in each sinus: top down behind the ring, and bottom up in front of the ring, effectively looping the ring. All sutures were passed 2 mm deep to the leaflet–aortic junction and were tied over fine Dacron pledgets, starting with the commissures. Each knot tower was fixed laterally to prevent abrasive contact with the leaflets.

**Figure 4:**
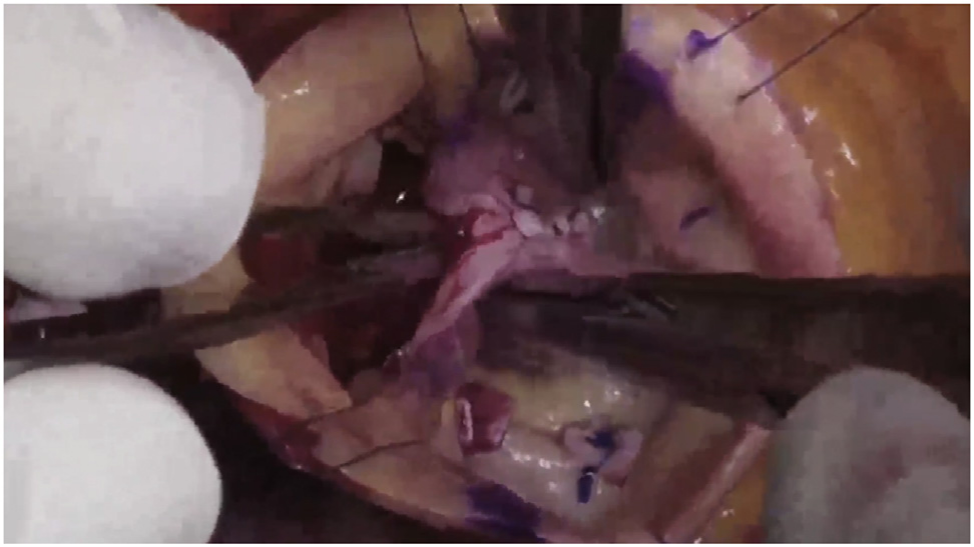
After repair, the annulus is reduced from 28 to 21 mm, the right prolapse is corrected with plications, and the noduli coapt centrally. With gentle “pressurization” using 3 closed DeBakey forceps, all 3 leaflets meet in the midline at the same coaptation height.

After ring placement, the leaflets met better in the midline, but as expected, the right coronary leaflet was prolapsing. Two free-edge plication sutures were placed symmetrically on either side of the right nodulus, and 2 more were required subsequently. Several of the noduli were rigid and noncompliant, so they were thinned with the ultrasonic aspirator. Then on testing, all 3 leaflets were at the same coaptation height and met well in the midline (Figure 4). Although the root enlargement was marginal (and aneurysm is not a usual feature of RCP syndrome), the larger noncoronary sinus of the asymmetric 46-mm root was excised, and a single 120° tongue of a 28-mm Valsalva graft (7 mm larger than the ring) was sutured to that sinus. The single suture line then was carried around the remaining aorta, and the distal anastomosis was completed. The combination of ring-based AVr and selective sinus replacement illustrated the versatility of these techniques.^4^^,^^5^ Postoperative echocardiography revealed no residual AI and a 5 mm Hg mean systolic gradient.

The second and third patients were presented to illustrate the consistent features of this syndrome. Both patients also were late middle-aged men with heart failure, severe AI, and dilated annuli. Echo findings were typical, with a posterior AI jet and RCP. After ring annuloplasty, symmetrical perinodular plication sutures were placed in the right coronary leaflet, and the prolapse was corrected. Ultrasonic nodular release also was required in both, and then the echocardiography showed good leaflet motion and competent valves (Video 1).

#### Video 2

To contrast with isolated RCP, Video 2 shows a 59-year-old man with recent AI onset and semiacute heart failure secondary to a ruptured fenestration and isolated noncoronary leaflet prolapse. His AI was severe with an anteriorly eccentric jet, together with minimal annular dilatation at 23 mm. On operative inspection, his noncoronary cusp prolapse was due to a ruptured fenestration to that leaflet. After placement of a 21-mm annuloplasty ring, the ruptured fenestration was sutured to the underlying leaflet with fine Prolene figure of 8 sutures to stabilize the commissure. The prolapsing noncoronary leaflet was plicated with symmetric perinodular Prolene sutures, achieving equal coaptation heights of >8 mm for all 3 leaflets. The post repair leak was trivial with a 5 mm Hg mean gradient.

#### Video 3

Two final patients with nodular scarring and retraction as the primary etiology of their AI are presented in Video 3. The first was a 51-year-old woman with congestive heart failure, a large ascending aortic aneurysm, and moderately severe AI. No significant leaflet prolapse was present, and the leaflet defect was related primarily to scarred retracted noduli (Video 3). The vena contracta was 6 mm, and on inspection, a central coaptation gap and scarred retracted noduli were evident. The small leaflets sized to a 19-mm ring. After ring insertion, all 3 noduli were gently thinned by 50% with the ultrasonic aspirator, and the leaflets then coapted well in the midline with equal free-edge heights. An ascending aortic graft was completed, and after repair, the residual leak was trivial with a mean valve gradient of 13 mm Hg.

The last patient was a 75-year-old woman with hypertension, left anterior descending stenosis, paroxysmal atrial fibrillation, and a large ascending aortic aneurysm with severe AI. The aneurysm narrowed at the sinotubular junction but extended into the aortic arch. The noduli were thickened, and the noncoronary leaflet was prolapsing. The annulus sized to 23 mm, but all 3 leaflets were 19 mm. After inserting a 19-mm annuloplasty ring, all 3 noduli were thinned by 50%, and the prolapsing noncoronary leaflet was plicated. At the end, all 3 leaflets met well in the midline with similar heights (Video 3). A left internal mammary artery to left anterior descending bypass graft was performed, the left atrial appendage was clipped, and the hemiarch was replaced using hypothermic circulatory arrest. After repair, the leaflets moved well with trivial residual leak and a mean pressure gradient of 15 mm Hg. The patient recovered without complications.

## Discussion

To our knowledge, this report is the first specific description of the syndrome of isolated RCP, despite the clinical frequency of this lesion as two-thirds of nonaneurysmal leaflet prolapse.^1^ Echocardiographic findings are characteristic, with a posterior AI jet and a “fractured” and “broken” right coronary leaflet tip (Figure 2). These new descriptive terms are appropriate to designate the unique pathology of RCP, but they also set this specific lesion apart from other types of leaflet prolapse. Most patients are late- to middle-aged men with a history of hypertension and exhibit a dilated annulus without an aneurysm, although classic findings might occur in aneurysm cases. Using geometric ring annuloplasty, valve repair becomes standardized. Leaflet plication requires a modest learning curve, and the presence of nodular scarring necessitates ultrasonic nodular release. The post-repair goal is achieving 3 supple Noduli Arantii coapting in the midline with equivalent coaptation heights of ≥8 mm.

Patients with isolated left or noncoronary prolapse are much less common and present differently—with less annular dilatation, an anterior AI jet, and frequent major leaflet defects (such as commissural or fenestration rupture). Management is similar with insertion of an annuloplasty ring appropriate for leaflet size and plication of the prolapsing leaflet. However, the commissural or fenestration rupture also requires management, usually with reconstruction using fine figure-of-8 Prolene sutures. Pericardial patches or polytetrafluoroethylene free-edge running sutures are avoided, because of a high incidence of late failure.^1^

Because nodular scarring and retraction are common, nodular thinning is key and produces good late results, as described previously.^1^^,^^3^^,^^6^^,^^7^ In most cases, leaflet plications are placed symmetrically, just lateral to the Noduli Arantii, to avoid nodular displacement away from the midline. Finally, overplication can pull the leaflets out of central coaptation and should be avoided by maintaining some commissural redundancy. Initial downsizing of the annuloplasty ring also can obviate consequent small central AI jets and facilitate reproducibility of the technique without increasing gradients.^3^

In previous studies of ultrasonic leaflet decalcification, a late incidence of cusp scarring, retraction, and AI were observed.^8^ However, these were heavily calcified valves, and proper annuloplasty ring stabilization was not available. In cases such as presented in this report, debridement and release of thickened Noduli Arantii have not resulted in late leaflet retraction,^6^^,^^7^ possibly because good leaflet coaptation height and support were re-established. The first cases of geometric annuloplasty were performed 10 years ago and included many patients with leaflet prolapse and nodular scarring.^1^ Recent Kaplan–Meier analysis including these patients showed a >80% survival and freedom from reoperation at 88 months of follow-up, consistent with standard repair-like outcomes.^9^ Thus, the benefits of valve repair appear to be accrued to patients with isolated RCP using the methods described.

## Conclusions

RCP without significant aneurysm is a common cause of AI. The defect is related to annular dilatation and subsequent “fracture” of the right leaflet tip. Similar findings can be observed in aneurysm patients, but less frequently. The management strategy of geometric ring annuloplasty, central leaflet plication, and nodular release is a straightforward and reproducible approach for durable repair (Video Abstract).

### Conflict of Interest Statement

Drs Wei and Rankin are or have been consultants for BioStable Science and Engineering, Inc, Austin, Texas. All other authors reported no conflicts of interest.

The *Journal* policy requires editors and reviewers to disclose conflicts of interest and to decline handling or reviewing manuscripts for which they may have a conflict of interest. The editors and reviewers of this article have no conflicts of interest.

## Supplementary Data

Video 1 Aortic valve repair for isolated right coronary leaflet prolapse. This video illustrates the clinical features and repair techniques for the syndrome of right coronary leaflet prolapse in 3 late- to middle-aged men with dilated aortic annuli and posterior AI jets. Video available at: https://www.jtcvs.org/article/S2666-2507(22)00146-8/fulltext.

Video 2 Aortic valve repair for isolated noncoronary leaflet prolapse using geometric ring annuloplasty. As a comparison with RCP, this video presents a patient with severe AI secondary to isolated noncoronary leaflet prolapse. The pathologic features are contrasted with RCP and repair techniques are illustrated. Video available at: https://www.jtcvs.org/article/S2666-2507(22)00146-8/fulltext.

Video 3 Ultrasonic nodular release during aortic valve repair for aortic insufficiency. For contrast to RCP syndrome, this video shows 2 cases of elderly women with small valves and ascending aneurysms, who also showed nodular scarring and retraction as part of the AI pathology. Techniques for repair of this entity are presented. Video available at: https://www.jtcvs.org/article/S2666-2507(22)00146-8/fulltext.

Video Abstract A 1-minute summary of the typical pathology of right coronary leaflet prolapse is provided, along with standardized techniques for aortic valve repair. Video available at: https://www.jtcvs.org/article/S2666-2507(22)00146-8/fulltext.
