# Supplementary figures and images for: Aortic valve repair for isolated right coronary leaflet prolapse
Source: JTCVS Tech. 2022 Mar 3;13:26–30. doi: 10.1016/j.xjtc.2022.02.031 (PMC9196763; doi:10.1016/j.xjtc.2022.02.031)

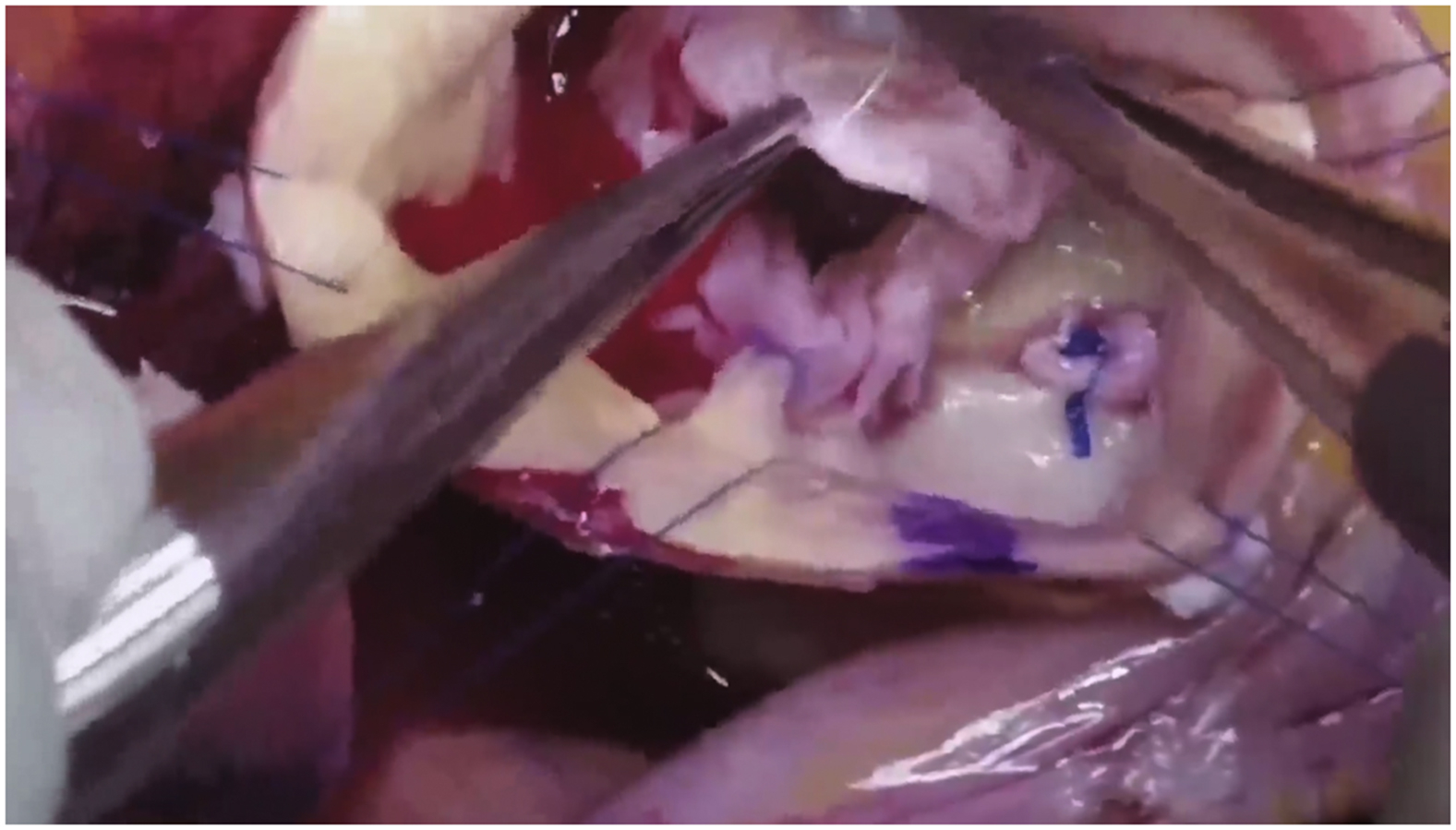

Supplement: Video 1 — Aortic valve repair for isolated right coronary leaflet prolapse. This video illustrates the clinical features and repair techniques for the syndrome of right coronary leaflet prolapse in 3 late- to middle-aged men with dilated aortic annuli and posterior AI jets. Video available at: https://www.jtcvs.org/article/S2666-2507(22)00146-8/fulltext. [file fx2.jpg]

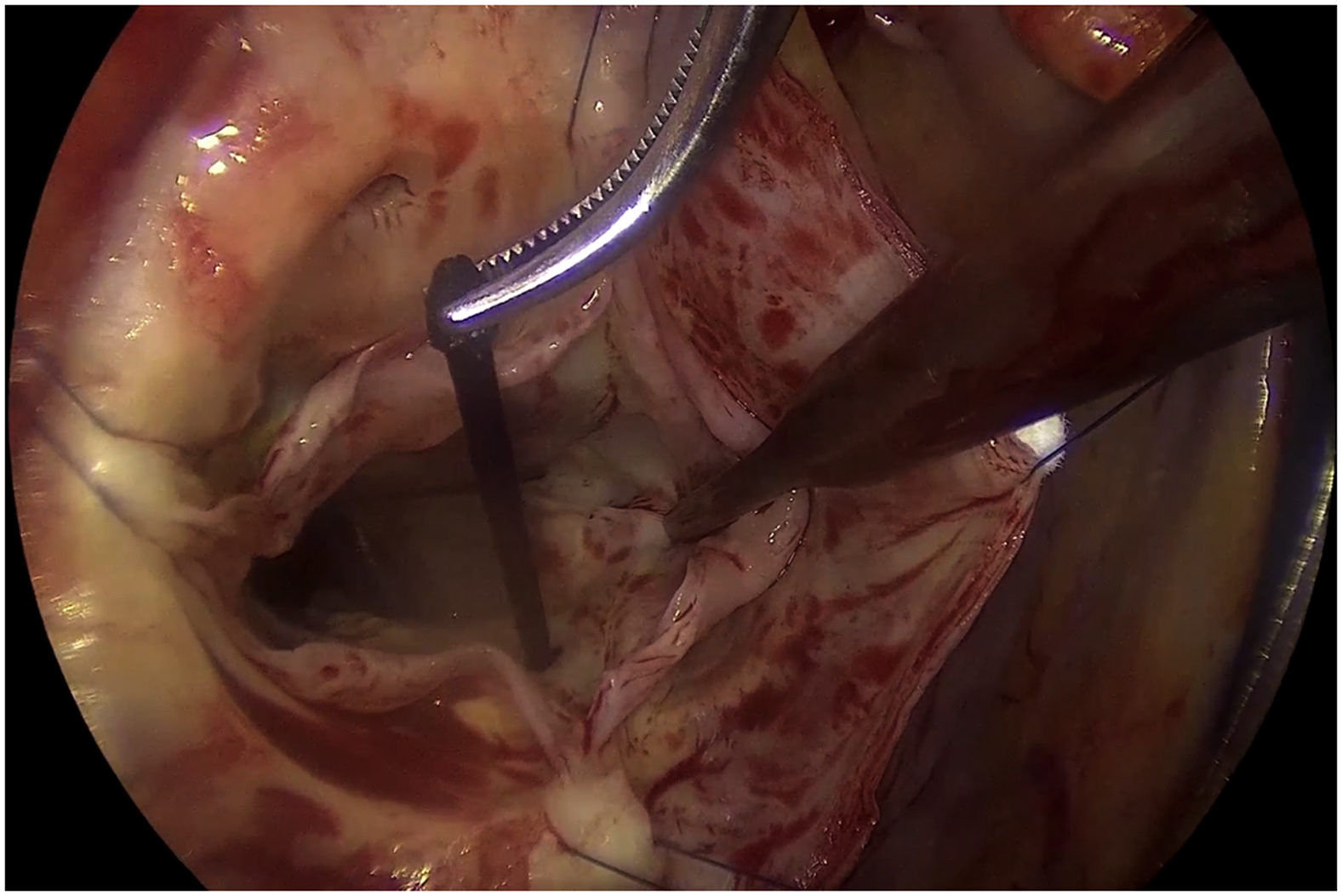

Supplement: Video 2 — Aortic valve repair for isolated noncoronary leaflet prolapse using geometric ring annuloplasty. As a comparison with RCP, this video presents a patient with severe AI secondary to isolated noncoronary leaflet prolapse. The pathologic features are contrasted with RCP and repair techniques are illustrated. Video available at: https://www.jtcvs.org/article/S2666-2507(22)00146-8/fulltext. [file fx3.jpg]

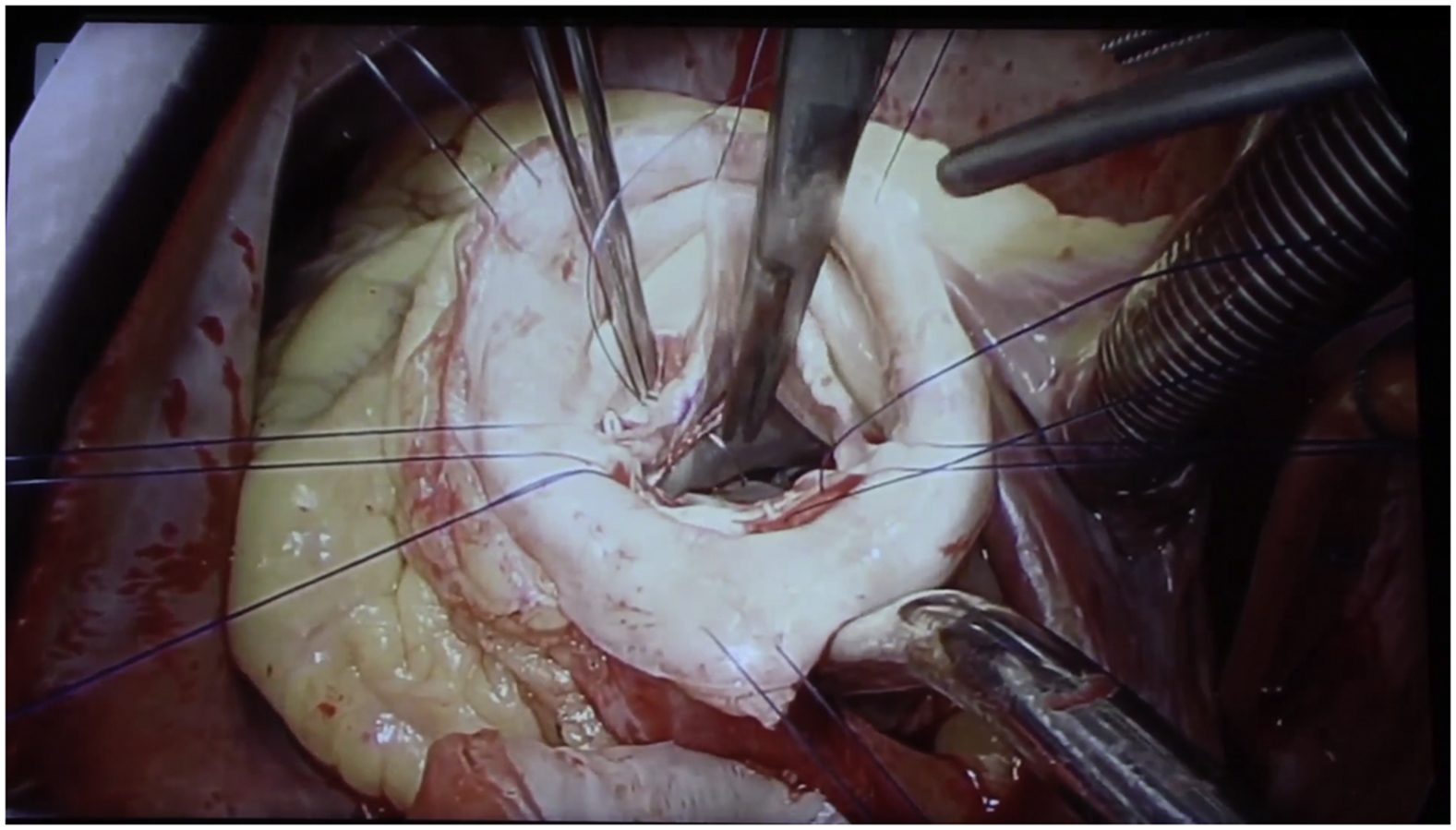

Supplement: Video 3 — Ultrasonic nodular release during aortic valve repair for aortic insufficiency. For contrast to RCP syndrome, this video shows 2 cases of elderly women with small valves and ascending aneurysms, who also showed nodular scarring and retraction as part of the AI pathology. Techniques for repair of this entity are presented. Video available at: https://www.jtcvs.org/article/S2666-2507(22)00146-8/fulltext. [file fx4.jpg]
